# Risk factors contributing to the possibility of conducting intensive home treatment and to the risk of hospitalization of 1045 home treated patients with Schizophrenia

**DOI:** 10.1192/j.eurpsy.2023.441

**Published:** 2023-07-19

**Authors:** A. Sabaté, R. Talisa, D. Córcoles, J. León, A. Malagon, A. M. González, M. Bellsolà, P. Samos, F. Casanovas, M. A. Jerónimo, L. M. Martin, V. Pérez

**Affiliations:** ^1^Psychiatry, Institut de Neuropsiquiatria i Addiccions, Parc de Salut Mar, Barcelona; ^2^Psychiatry, Xarxa de Salut Mental i Addiccions de la Provincia de Girona, Institut d’Assistència Sanitària (IAS), Parc Hospitalari Martí i Julià, Salt, Girona, Spain

## Abstract

**Introduction:**

Home Treatment (HT) teams are among the better-studied options to reduce admission at the hospital, having been described as an alternative to hospitalization in patients with schizophrenia. There may be certain risk factors which has already been described such as living alone (Dean and Gadd, BMJ, 1990; 301, 1021–1023; Schnyder et al., Acta Psychiatr. Scand. 1999; 99, 179–187), lack of awareness of the illness, uncooperativeness (Cotton et al., BMC Psychiatry, 2007; 7, 52) and fewer visits carried out (Morgan et al., Aust. New Zeal. J. Psychiatry,2006; 40, 683–690) which together can negatively influence the possibility of conducting intensive home follow-up and, therefore, increase the likelihood of hospitalization.

**Objectives:**

To describe de relative contribution of several risk factors to patient hospitalization related to the possibility of conducting intensive home follow-up of patients diagnosed with Schizophrenia following home treatment. Second, to determine de risk of hospitalization related to the possibility of conducting intensive home follow-up according to the presence of one or more risk factors of patients diagnosed with Schizophrenia following home treatment.

**Methods:**

All patients with schizophrenia who were visited by a home treatment team in Barcelona between January 2017 and December 2021 were included in the study. To assess whether there was an increased risk of hospitalization associated with factors such as living alone, uncooperativeness (PANSS G8 item >= 4) and ≤1 home visit, two bivariate logistic regression analyses were conducted. We studied these factors as independent variables to assess the relative contribution to the risk of hospitalization, and we studied if the presence of 1, 2, 3 or 4 of these risk factors as independent variables worsened the risk of hospitalization.

**Results:**

Uncooperativeness shows the highest contribution to the risk of hospitalization, followed by ≤ 1 home visit, lack of insight and living alone, all results reaching significance (p=0.000).

There is an increase in the risk of hospitalization depending of the presence of 1,2,3 or 4 of these risk factors (1 risk factor (Odds Ratio = 1.21), 2 risk factors (Odds Ratio = 5.28), 3 risk factors (Odds ratio = 13.53), 4 risk factors (Odds ratio = 29.18).

**Conclusions:**

There are a number of factors directly related to the possibility of conducting intensive follow-up that appear relevant in the case of psychotic patients in acute crisis treated at home. This set of variables are the lack of awareness of the illness, lack of collaboration, living alone and the number of visits that have been made, all with statistically significant differences in our study. These factors together also greatly increase the risk of hospitalization, becoming almost 30 times more likely when these 4 factors are present.

**Disclosure of Interest:**

None Declared

